# Optimal coordinated voltage control in active distribution networks using backtracking search algorithm

**DOI:** 10.1371/journal.pone.0177507

**Published:** 2017-10-09

**Authors:** Tengku Juhana Tengku Hashim, Azah Mohamed

**Affiliations:** 1 Department of Electrical Power Engineering, Universiti Tenaga Nasional, UNITEN, Kajang, Malaysia; 2 Department of Electrical, Electronic and Systems Engineering, Universiti Kebangsaan Malaysia, Bangi, Malaysia; Nankai University, CHINA

## Abstract

The growing interest in distributed generation (DG) in recent years has led to a number of generators connected to a distribution system. The integration of DGs in a distribution system has resulted in a network known as active distribution network due to the existence of bidirectional power flow in the system. Voltage rise issue is one of the predominantly important technical issues to be addressed when DGs exist in an active distribution network. This paper presents the application of the backtracking search algorithm (BSA), which is relatively new optimisation technique to determine the optimal settings of coordinated voltage control in a distribution system. The coordinated voltage control considers power factor, on-load tap-changer and generation curtailment control to manage voltage rise issue. A multi-objective function is formulated to minimise total losses and voltage deviation in a distribution system. The proposed BSA is compared with that of particle swarm optimisation (PSO) so as to evaluate its effectiveness in determining the optimal settings of power factor, tap-changer and percentage active power generation to be curtailed. The load flow algorithm from MATPOWER is integrated in the MATLAB environment to solve the multi-objective optimisation problem. Both the BSA and PSO optimisation techniques have been tested on a radial 13-bus distribution system and the results show that the BSA performs better than PSO by providing better fitness value and convergence rate.

## 1 Introduction

**Fig 1:**
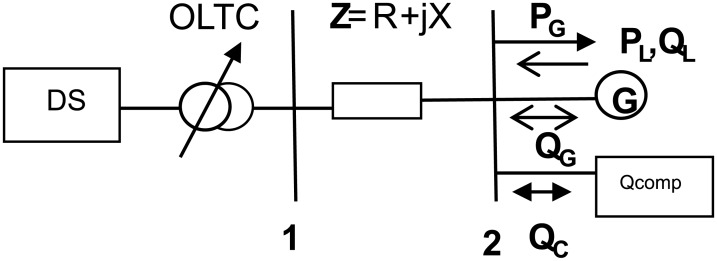
A simple system connected with DG.

Distributed generation (DG) is the integrated use of small-capacity generators that are directly connected to a distribution system at distribution-end level and closer to customers [1]. The increasing penetration of DGs leads to technical issues because DGs interact with the existing elements of traditional distribution systems. Some technical issues that are generated from the existence of DGs in a network include voltage levels, power flows, equipment thermal ratings, fault current levels, and protection issues [2]. Voltage control is a well-documented operational challenge drawn from the literature review [3]. Distribution network operators consider three worst-case operating scenarios associated with voltage issues in ensuring that customers and networks are not adversely affected. These scenarios are categorised as; i) no generation and maximum power demand, ii) maximum generation and minimum system demand, and iii) maximum generation and maximum system demand [4]. Increased generation reverses power flow along the line from the generator to the substation, in which voltage rise becomes severe in the absence of demand because all the generation is exported back to the primary substation. As a result, developing a stable and well-coordinated voltage control scheme is essential to deal with the voltage rise issue. The voltage rise issue is illustrated in Fig 1, in which a simple two-bus distribution system is connected with DG.

In this simple system, the generator G with generation P_G_, Q_G_ together with local load, P_L_, Q_L_, and a reactive compensator Q_C_, is connected to the distribution system through a weak rural overhead line with impedance Z and a transformer with an on-load tap-changer (OLTC). From the figure, the approximate voltage at bus 2 (V_2_) can be calculated as follows:

$$V_{2}=V_{1}+R(P_{G}+P_{L})+(Q_{G}+Q_{L}+Q_{C})X$$ (1)

The above equation can be used to qualitatively analyse the relationship between the voltage at bus 2 and the amount of generation that can be connected. Based on the equation, the impact of alternative control actions on managing voltage rise can also be analysed. Voltage level is particularly influenced by the following factors: (i) line resistance R, (ii) line reactance X, (iii) DG power output (P_G_, Q_G_), (iv) reactive power compensator Q_C_, (v) local load (P_L_, Q_L_), and (vi) voltage at bus 1 (V_1_). These factors serve as the basic elements for the options of voltage control to manage voltage rise issues in distribution networks connected to DG.

With reference to [5], active network management schemes are currently classified as centralised or coordinated schemes and decentralised or distributed control schemes. Centralised or coordinated control strategies provide voltage regulation from the substation to the rest of the network by potentially using a wide range of communication systems to coordinate different devices in the systems, such as OLTCs and voltage regulators. Examples of coordinated voltage control methods include the implementation of distribution management systems to coordinate the distribution network components using intelligent coordinated methods [5]. On the other hand, semi-coordinated and decentralised or distributed control strategies control the DG unit locally in an active manner while coordinating it with a limited number of other network devices. Examples of decentralised voltage control methods include power factor control; OLTC control; reactive power compensation using reactive power control devices, such as shunt capacitors or static Var compensators; generation curtailment; and decentralised intelligent methods, which are implemented to provide automation to the control methods.

Considerable amount of research has been done using intelligent techniques for optimising the settings of control methods, otherwise known as heuristic optimisation techniques in coordinated voltage control. These optimisation techniques include the use of genetic algorithm (GA), particle swarm optimisation (PSO), ant colony algorithm (ACO), evolutionary programming, Tabu search, and honey-bee mating optimisation. One of the early optimisation technique is the GA, which has been widely used for solving power system optimization problems, specifically, voltage control problem as carried out in this study. In a research work carried out in [6], the voltage profile was found to be improved and the network losses was reduced by reconfiguration and sectionalising the 33-bus radial distribution system using GA as the optimisation tool. In another work, GA was used to calculate the optimal setting of a voltage regulator under various load conditions in a 13-bus unbalanced radial distribution system with the objective of keeping voltages at the nodes within allowable limits [7]. A fuzzy-based GA was developed for voltage control on a realistic distribution system that is part of the North Delta Electricity Distribution Company network in Egypt [8]. In this work, objective function and constraints were formulated in terms of fuzzy sets, and GA was adopted to determine the optimal dispatching schedule of the main transformer tap position and the on/off status of the capacitors. In [9], a GA derivative known as non-dominated sorting genetic algorithm was employed to determine the coordinated optimal settings of OLTC, reactive power, and active power in minimizing losses, voltage deviation, and fault critical clearing time of the system. Another work was tested on the 33-bus radial distribution system, in which an improved GA was used to find the optimal values of two voltage control methods of DG, which are active power output and reactive power compensation position [10]. In [11], a GA-based centralised voltage control strategy was proposed and tested on the modified IEEE 14-bus test system and on the distribution system in the northern part of Thailand. The objective of this work was to minimise the steady-state voltage fluctuations and to maintain the system voltage profile within permissible range by coordinating the operations of voltage/reactive power control equipment, such as OLTCs, shunt capacitors, and automatic voltage regulators. On the other hand, PSO is another popular optimisation technique that is widely used in a wide range of optimisation problems related to voltage control. In [12], PSO was used to determine an offline day-ahead planning to determine the optimal settings of all control devices, including OLTCs and switched shunt capacitors. The results from the research showed that voltage profile, losses, and costs were improved. In another work [13], PSO was used on the modified 29-bus distribution system of the Thailand’s Provincial Electricity Authority. The findings of this case study revealed that the optimal coordination between the under-load tap-changer and the substation and feeder capacitors for volt/Var control was able to minimise the costs and energy loss. In [14], a new fuzzy adaptive PSO method was applied and tested in a distribution system with DGs to determine the optimum active and reactive power dispatch for DG units which includes the reactive power contribution of capacitor banks and the tap settings of transformers a day in advance. A multi-objective adaptive PSO algorithm for reactive power optimisation and voltage control was implemented in [15]. PSO was improved by adaptively adjusting the inertia weight to avoid premature convergence. PSO was also implemented in a distribution management system to compute the optimised reactive power set points of the DGs in a distribution system [16].

ACO is an optimisation technique for solving graphical and combinatorial optimisation problems. It is based on a multi-agent system that utilises the behavior of each single agent, in this case, the artificial ant, which is inspired by the behavior of real ants [17]. In [18], ACO was applied and integrated with DGs in a distribution management system to determine the optimal settings of tap changers, capacitor, and reactive power of DGs based on the state estimation output of the system. ACO was also combined with another technique of Delaunay triangulation in a medium-voltage network planning to find optimal power factor values for the DGs [19]. This combined method was found to be able to reduce the network costs to about 2.5% lower compared to the costs by using conventional planning. A binary ant colony optimisation (BACO) was tested on the IEEE 33-bus and 69-bus radial distribution systems and used to determine the optimum dispatch schedules for OLTC, substation switched capacitors, and feeder switched capacitors based on a day-ahead load forecast [20]. In this research work, a multi-objective function for minimising voltage deviation, total electrical energy loss, number of OLTCs and capacitors, and voltage fluctuations was formulated. Accordingly, BACO was found to perform better compared with other optimisation techniques of binary genetic, genetic, hybrid, and PSO algorithms. Another optimisation technique associated with voltage control in distribution networks with DGs is the honey-bee mating optimisation technique, which is implemented to determine the optimal values of active power of DGs, reactive power of capacitors, and tap positions of transformers [21]. This technique was improved by incorporating chaotic theory and fuzzy logic and was tested on the IEEE 69-bus test system to achieve minimal cost, electrical energy loss, and voltage deviation. An improved and nested evolutionary programming approach was presented in [22] to assign voltage control variables, such as voltage set points at the HV/MV substation, as well as tap-changer positions and voltage set-points of local generators to minimise voltage deviations at load buses.

In this research work, the aim is to find the optimal settings of the power factor and OLTC for the coordinated voltage control in a distribution system with DGs. The amount of generation to be curtailed is also determined by using a relatively new optimisation technique called as the backtracking search algorithm (BSA) [23]. Three different case studies with different levels of power generations are used to test the BSA on the radial 13-bus distribution test system. BSA is also compared with PSO and the results show that BSA performs better with faster convergence rate and fitness function. The BSA also gives better accuracy in reducing voltage deviation and active power losses in the system.

## 2 Problem formulation

A multi-objective optimisation technique, formulated as a constrained nonlinear integer optimisation problem, is used to determine the optimal settings of power factor for DG, the OLTC, and the amount of active power to be curtailed. The objective functions are to minimise total power loss and voltage deviation at load buses. The fitness function can be expressed as follows:

$$F_{\text{min}}=\alpha (P_{loss})+\beta (V_{dev})$$ (2)

where *F* is the fitness function, *P*_*loss*_ is the total power loss (%), *α* is the coefficient factor for total power loss, *V*_*dev*_ is the voltage deviation (%) at all system busbars, and *β* is the coefficient factor for *V*_*dev*_. Total real power loss is defined by the following equation:

$$P_{loss}=\sum_{i=1}^{n}\;P_{loss_{i}}i=1,2,3\ldots n$$ (3)

where *n* is the number of lines. *V*_*dev*_ is defined as

$$V_{dev}=\frac{V_{iref}-V_{i}}{V_{i}}$$ (4)

where *V*_*iref*_ is the reference voltage at bus *i* and *V*_*i*_ is the actual voltage at bus *i*.

The total power loss and voltage deviation should be minimised according to the network power flow equations. Generally, multi-objective function methods provide a set of optimal solutions. The best optimisation solution is obtained by using the sum of the coefficient factor method. The coefficient factor for total power loss is assumed to be 0.6 while the voltage deviation is considered to be 0.4. The reason behind the factor for power loss is greater than voltage deviation is due to the fact that the reduction of power loss in distribution networks gives more impact on economic and technical prospects of the power system.

Inequality constraints are the constraints which are related to the bus voltages, in which the bus voltage magnitudes must be kept within acceptable operating limits throughout the optimisation process. The bus voltage constraint is defined as follows:

$$V_{\text{min}}\leq \left|V_{i}\right|\leq V_{\text{max}}$$ (5)

where *V*_min_ is the lower bound of bus voltage limits, *V*_max_ is the upper bound of voltage limits, and | *V*_*i*_ | is the root-mean-square value of the *i*^th^ bus voltage.

### 3 Backtracking search algorithm

BSA is a new evolutionary algorithm (EA) developed by Pinar Civicioglu in 2013 to solve real-valued numerical optimisation problems. EA is a popular stochastic search algorithm that is widely used to solve nonlinear, non-differentiable, and complex numerical optimisation problems. It is based on stochastic search mechanisms that search for near-optimal solutions to a problem. The most commonly used EA optimisation techniques are based on swarm intelligence and genetic evolution. The new EA method known as BSA is unique in terms of its mechanism to generate a trial individual, which enables it to solve numerical optimisation problems successfully and rapidly. BSA can be explained by dividing its functions into the following processes: initialisation, selection-I, mutation, crossover, and selection-II. As described in detail in [23], the computational procedures of the BSA technique are as follows:

1. BSA initialises the population P using the following equation:
  $$P_{i,j}\sim U\left(low_{j},Up_{j}\right)$$ (6)
  For *i* = 1,2,3,…, *N* and *j* = 1,2,3…, *D*, where *low*_*j*_, *up*_*j*_ are the lower and upper limits of the problem dimension, while *N* and *D* are the population size and the problem dimension respectively, *U* is the uniform distribution, and each *P*_*i*_ is a target individual in the population *P*.
2. BSA selection-I stage determines the historical population *oldP* to be used for calculating the search direction. The initial historical population is determined by the following equation:
  $$oldP_{i,j}=U\left(low_{j},up_{j}\right)$$ (7)
  BSA has the option of redefining *oldP* at the beginning of each iteration through the “if-then” rule as in Eq (8).
  ifa<bthenoldP≔P|a,b~U(0,1), (8)
  where: = is the update operation and P|*a*, *b* ~ *U*(0, 1) is a Boolean-valued function that returns true only values, for example 0 = false and 1 = true. Eq (8) ensures that BSA designates a population belonging to a randomly selected previous generation as the historical population until it is changed. This condition allows BSA to acquire a memory. After the *oldP* is determined, Eq (9) is used to randomly change the order of the individuals in *oldP*.
  oldP≔permuting(oldP) (9)
  The permutation function used in Eq (9) is a random shuffling function.
3. The mutation process of BSA generates the initial form of the trial population *Mutant* by using Eq (10).
  Mutant=P+F×(oldP−P) (10)
  where *F* controls the amplitude of the search-direction matrix (*oldP-P*). BSA generates a trial population while taking partial advantage of its experience from previous generations so that the historical population is used in calculating for the search-direction matrix. The value for *F* = 3.*rndn whererndn ~* N (0, 1), where *N* is the standard normal distribution.
4. The crossover process of BSA generates the final form of the trial population *T*. The initial value of the trial population is *Mutant*, as set in the mutation process. Trial individuals with better fitness values for optimisation are used to evolve the target population individuals. The crossover process has two steps. The first step calculates a binary integer-valued matrix (map) of size *N*.*D*, which indicates the individuals of T to be manipulated by using relevant individuals of *P*. If *map*_*n*,*m*_ = 1, where *n* ∈ {1, 2, 3, …, *N*} and *m* ∈ {1, 2, 3, …, *D*}, *T* is updated with *T*_*n*,*m*_ ≔ *P*_*n*,*m*_. The unique crossover strategy of BSA is different and more complex compared with the crossover strategies used in differential equation and its variant. Some individuals that overflow the allowed search space limits as a result of the mutation strategy of BSA are also regenerated. The difference in the unique crossover strategy and the regenerated individuals are shown in Algorithms 2 and 3 respectively (23).
5. In the BSA selection-II stage, the T_*i*_s that have better fitness values than the corresponding P_*i*_s are used to update the P_*i*_s based on a greedy selection. If the best individual of *P* (P_*best*_) has a better fitness value than the global minimum value obtained so far by BSA, the global minimizer is updated as P_*best*_ and the global minimum value is updated as the fitness value of P_*best*_.

BSA is unique and different from the other algorithms in terms of its mutation, crossover, and boundary control mechanisms. Moreover, it is a dual-population algorithm that uses both current and historical populations and has a much simpler structure than the other algorithms.

**Fig 2:**
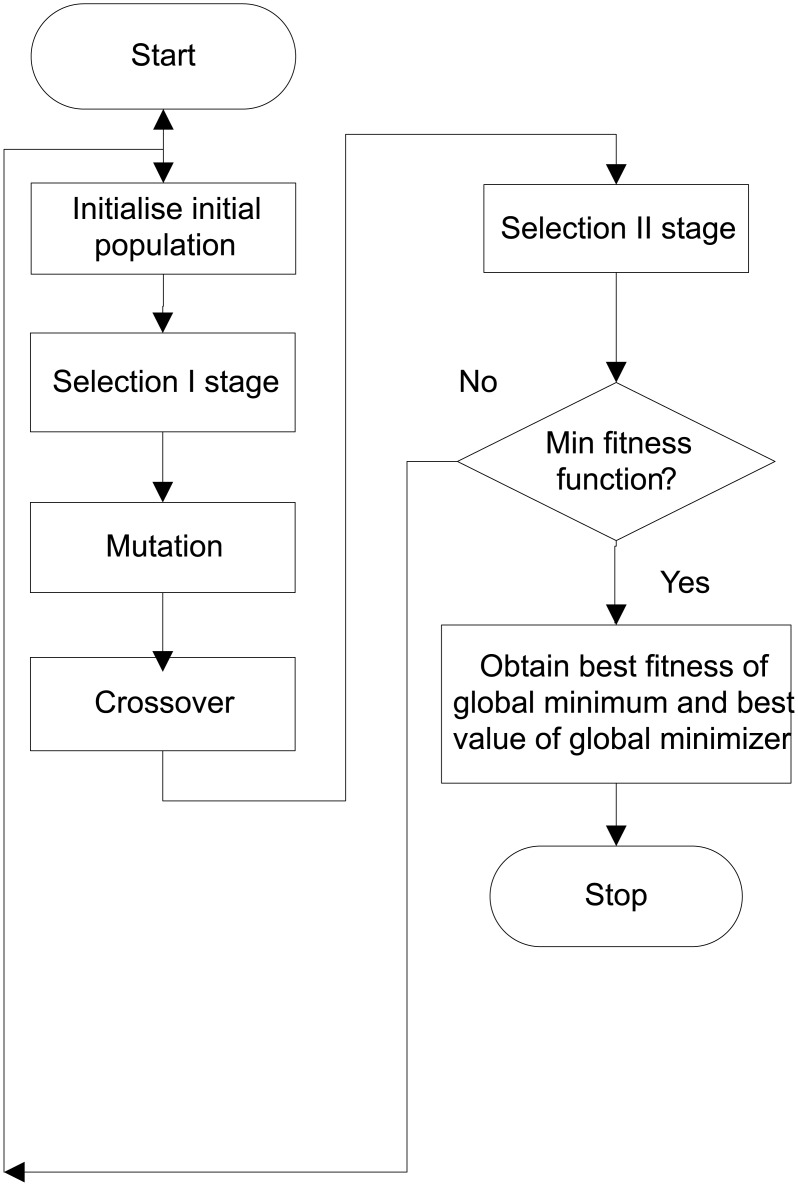
Flowchart of the BSA optimisation technique.

**Fig 3:**
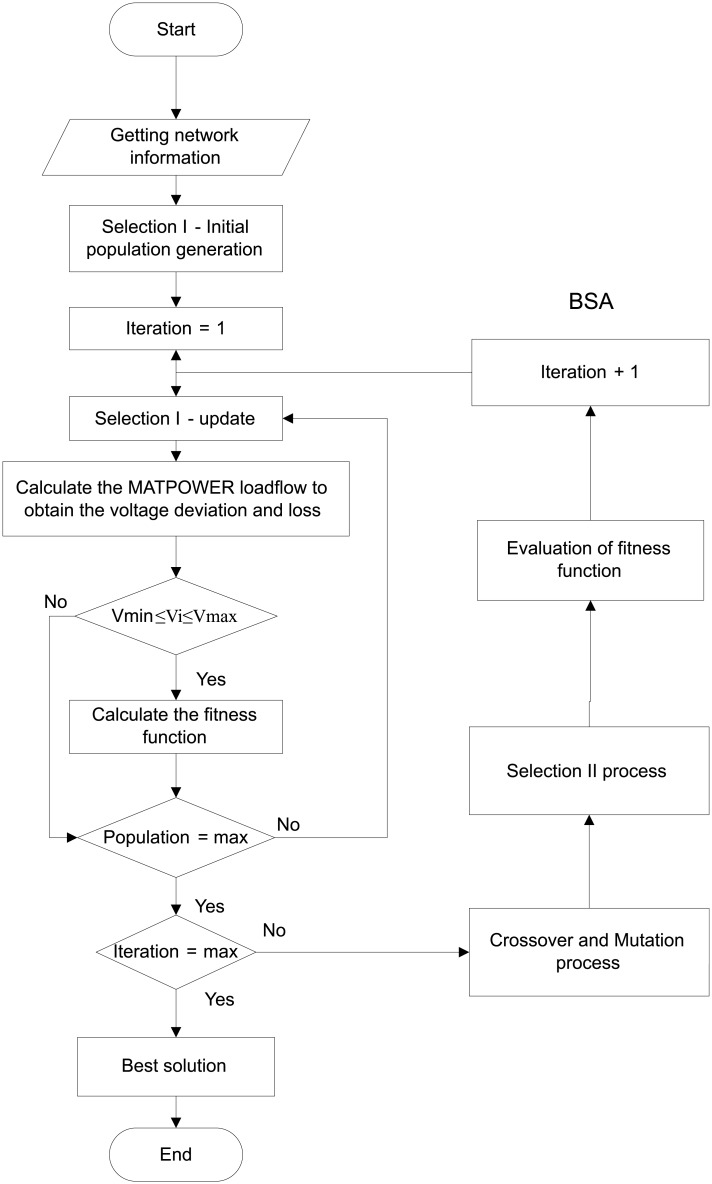
Flowchart for the BSA algorithm for determining the optimal settings of the coordinated voltage control in a distribution system with DGs.

Fig 2 depicts the general flowchart of BSA, whereas Fig 3 shows the detail flowchart of the implementation of the BSA technique for determining the optimal settings of the three voltage control methods using PFC, OLTC and generation curtailment as well as to minimise the losses and voltage deviation.

The detailed steps of the flowchart in Fig 3 are described as follows:

1. Obtain the input network information such as bus, line and generator data.
2. Randomly generate the initial population as in Selection I stage within the population size and dimension. Set the feasible solution combination, such as the PFC settings to be within 0.85 to 0.95, OLTC settings within 0.98 p.u to 1.03 p.u and the amount of generation to be curtailed for both DGs to be within 0% to 40%.
3. Run the MATPOWER loadflow to determine the total power loss and voltage deviation.
4. Check the bus voltage magnitude constraints. If the limit is exceeded, repeat step iii.
5. Calculate the fitness function using Eq 2.
6. Perform the mutation process to generate the initial form of the trial population *Mutant* by using Eq 10.
7. Create the final form of the trial population, T using the crossover process.
8. Implement the Selection II stage, where the population P_i_s which has better fitness value than the target Tis are used to update the P_i_s. From the selection process, if the minimum fitness function is achieved, then the best fitness function called as the global minimum with the best optimal values known as the global minimiser is achieved and selected as the solution.
9. Repeat the process until the stopping criteria is met and the best solution is obtained.

## 4 Particle swarm

**Fig 4:**
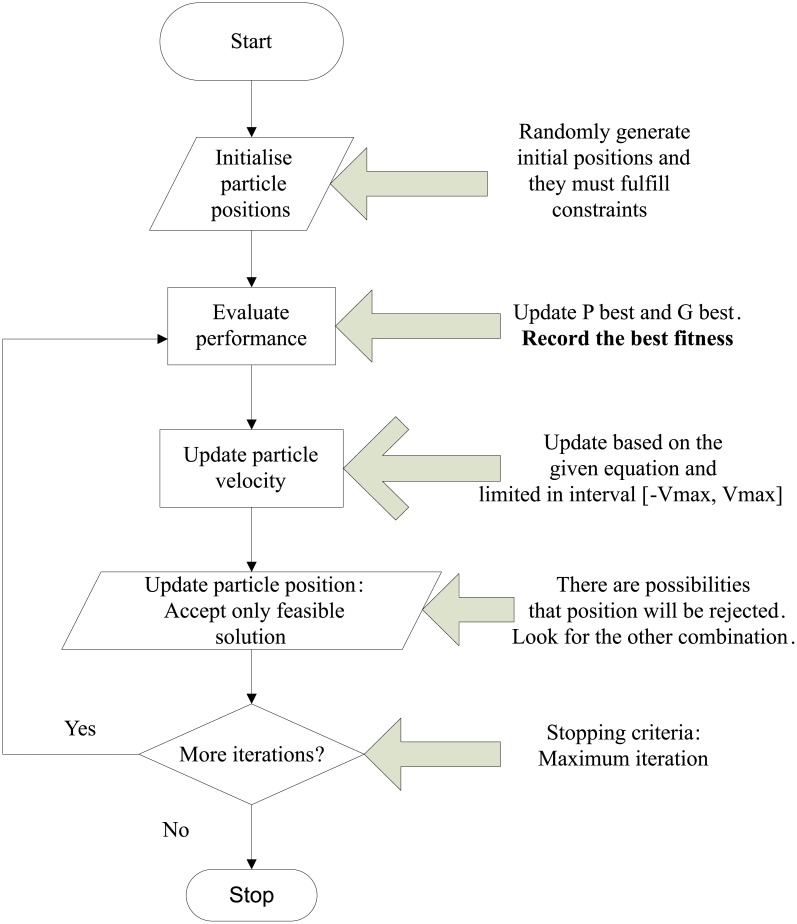
General steps of PSO.

PSO was first developed by Kennedy in 1995 [24]. It is a population intelligent optimisation algorithm that has gained considerable attention from scholars in various fields for more than a decade. Unlike GA, PSO was inspired by the choreography of a bird flock, which can be regarded as a distributed behavior algorithm that performs multidimensional search. Birds and other swarm creatures, such as fish, adjust their physical movement to avoid predators, seek food and mates, and optimise environmental parameters such as temperature. The term “particle” was selected as a compromise instead of particular organisms, such as fish or bird. Each particle is represented by its position and velocity, and is referred to as a potential solution in the *n*-dimensional search space of the problem. To find a good optimum, PSO conducts a search through the interaction of particles in the swarm by exploring different regions of the search space. In general, the PSO algorithm consists of three major steps: i) generate the initial particle’s positions and velocities, ii) evaluate the fitness value of each particle, and iii) update the velocity and position of all particles [25]. The important processes in PSO are described and shown in Fig 4.

## 5 Implementation of BSA and PSO optimisation techniques for optimal coordinated voltage control

**Fig 5:**
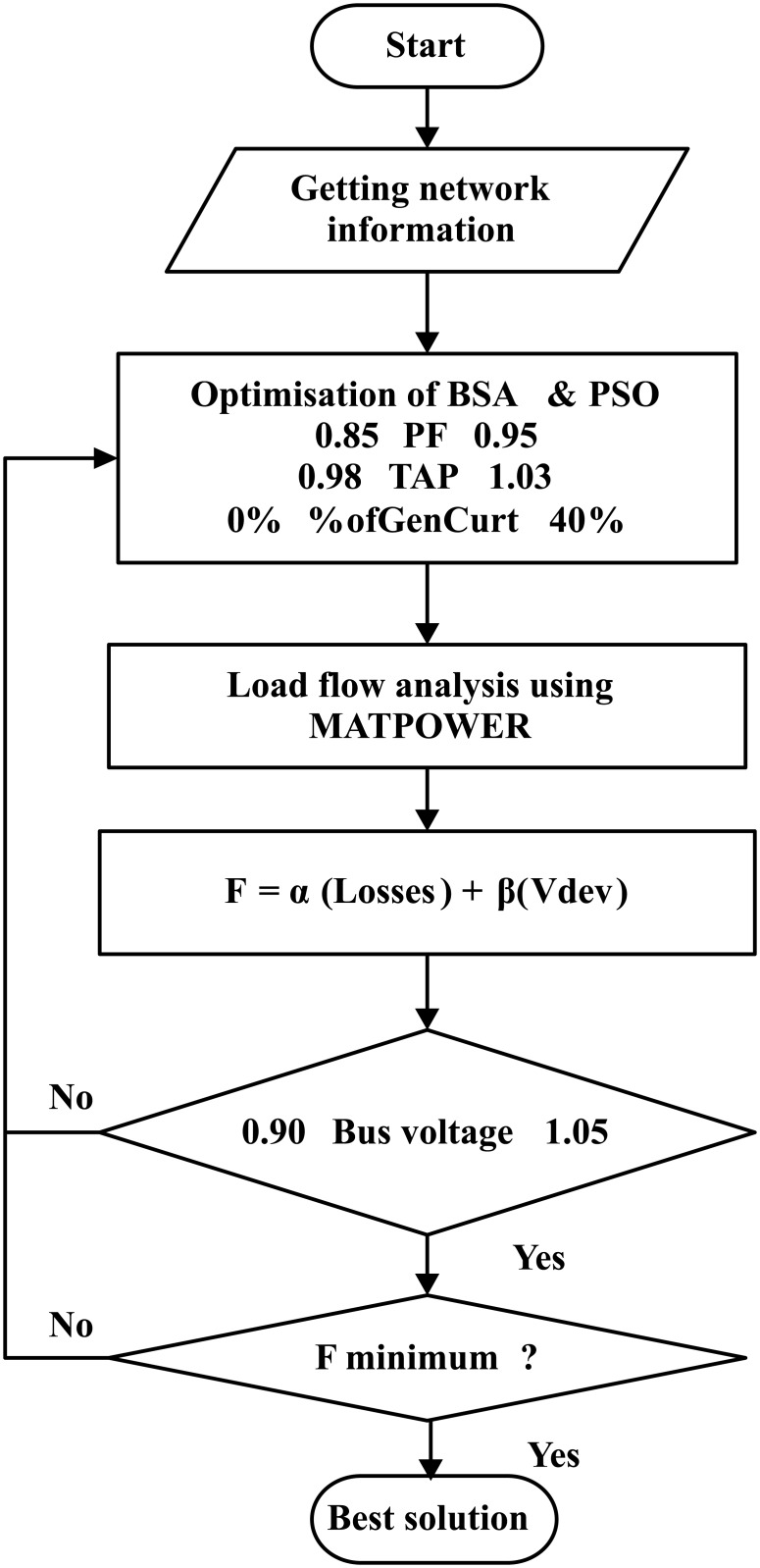
Procedure of optimal coordinated voltage control using BSA or PSO.

Fig 5 shows the flowchart of the optimisation procedures which utilise BSA or PSO as well as the MATPOWER Newton-Raphson load flow analysis. Based on the flowchart, the first process is to obtain the distribution network information, which includes the parameters to be optimised. The next step is to set the boundary constraints for the parameters to be optimized. Then, MATPOWER is used to perform load flow analysis, and is integrated into the MATLAB coding. The objective function is set to minimise power loss and voltage deviation of the system. The voltage limits of the system must be within its permissible limits. Once the minimum fitness function is obtained, the optimisation solution is achieved, in which the value is taken as the best solution.

## 6 Simulation results

**Fig 6:**
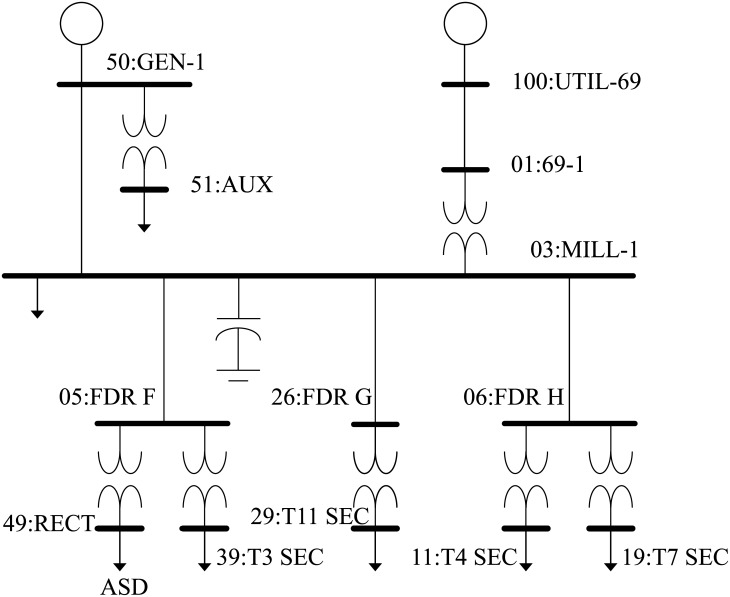
13-bus radial distribution system.

Both the BSA and PSO techniques have been applied for determining the optimal coordinated voltage control settings in the 13-bus balanced radial industrial distribution system (Fig 6). The load and bus data of the system are given in [25] and the system loads are considered as spot loads with a total of 9.28 MW and 7.58 MVAr. The minimum and maximum voltage limits are set at 0.9 and 1.05 p.u., respectively and the maximum iteration for the BSA and PSO algorithms are set at 1000. Both the algorithms are coded and implemented in the MATLAB computing environment.

The 13-bus radial distribution system consists of DGs which are synchronous machine-based DGs with a capacity range of 1 MW to 3 MW. The total number of DGs installed in the system is two and are located at buses 03:MILL-1 and 39:T3 SEC respectively. Altogether there are seven tap changers in the test system. Through the coordinated voltage control actions of power factor (PF), tap changer, and generation curtailment, the optimal settings of the three voltage control methods mentioned are determined to minimise voltage deviation and losses of the system. Several assumptions are made for the performed optimisation:

1. The DG will only inject active power.
2. The number of DGs connected to the system is two.
3. The base case system is free from any DG connection. DG has no contributions to the voltage profile and losses of the system.

The boundary constraints for the control parameters, such as power factor, tap changer, and amount of generation to be curtailed, are as follows:

1. 0.85 ≤ PF ≤ 0.95
2. 0.98 ≤ tap settings ≤ 1.03
3. 0 ≤ % of MW generation ≤ 40

Three cases are considered, in which a total number of two DGs with capacities of generations are varied to test the effectiveness and consistencies of the optimisation techniques under investigation. The amount of power of the DGs are increased for i) 1 MW, ii) 2 MW, and iii) 3 MW. The variables and components of interest, which include voltage profiles, system losses, voltage deviation at load buses, and optimal values of the coordinated voltage control settings, are recorded and compared for both the optimisation techniques applied.

**Fig 7:**
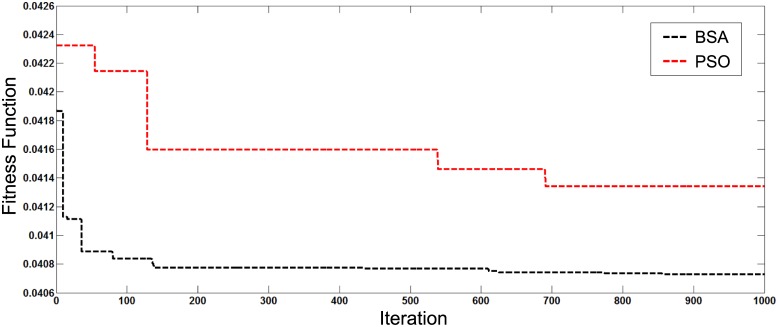
Convergence characteristics of BSA and PSO for DGs with 1 MW generation.

**Fig 8:**
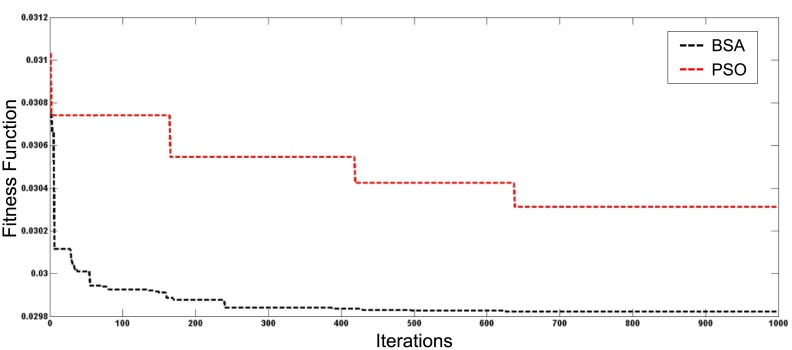
Convergence characteristics of BSA and PSO for DGs with 2 MW generation.

**Fig 9:**
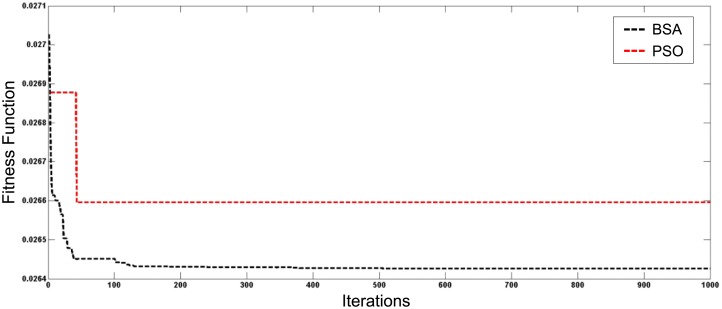
Convergence characteristics of BSA and PSO for DGs with 3 MW generation.

Fig 7 depicts the best fitness value among 30 simulation runs for both optimisation techniques for the first case of total generation of 1 MW of DGs installed in the distribution system. Figs 8 and 9 show the best convergence characteristics of the system with generations increased to 2 MW and 3 MW, respectively. Results indicate that the BSA gives better fitness and convergence rate compared to PSO.

**Fig 10:**
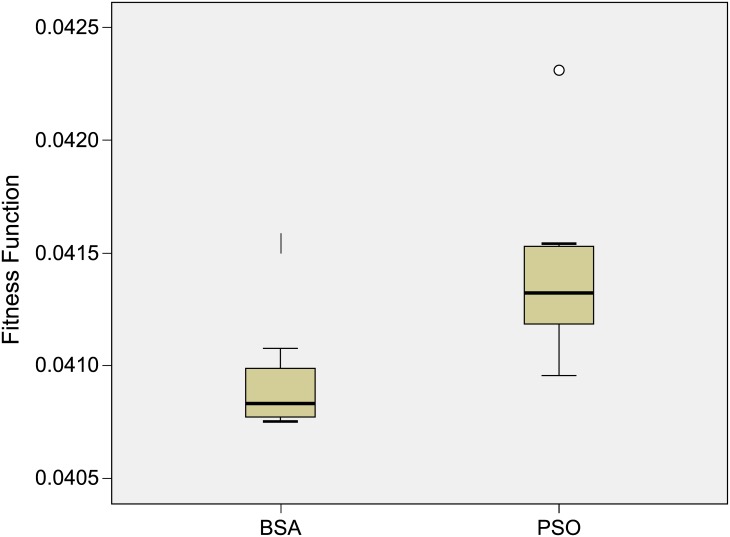
Box and whiskers plot representation of the fitness function obtained using BSA and PSO for 3MW case.

Fig 10 shows a sample of the box and whiskers plot of the distribution of the fitness function obtained using both the BSA and PSO techniques for the 3MW case. From the plots, it can be seen that the distribution of the fitness function data for both BSA and PSO are more skewed to the upper quartile. The median of the data for BSA is approximately 0.0407 and for PSO the median of the value obtained is approximately 0.0413. The maximum outlier for the fitness function data distribution obtained using PSO is approximately 0.0423 whereas for BSA the value is around 0.0416. From the plots, it is clearly seen that BSA performs better in terms of fitness function compared to PSO by obtaining lower values of data distribution.

Considering the consistency and robustness of the developed optimisation techniques, 30 independent runs were performed for each case to measure the frequency in reaching the optimal or near-optimal solutions while maintaining the same stopping criterion (maximum iteration of 1000). Results are shown and summarised for DGs connected to the test system with total power generations varying from 1, 2, and 3 MW. For all cases, the BSA technique obtains better solution in terms of fitness function. Tables 1 to 4 summarise the optimal values of the control variables in terms of power factor and tap-changer settings, and the percentage of generation amount to be curtailed in the test system for the three different case studies. The objective functions of this study are to minimise the power loss and voltage deviation of the system. The overall optimisation results are tabulated as shown in Table 4. The base case power loss is found to be 0.146 MW, whereas average voltage deviation for the base case is 0.014 V. The overall performance of the system shows a decreasing percentage in power loss and voltage deviation with optimal DG parameter settings.

**Table 1:** Optimisation result with PSO and BSA for DGs with 1 MW generation of the 13-bus test system.

	PSO	BSA
Best fitness	0.0266	0.0264
Tap DG1	0.9935	0.9802
Tap DG2	1.0284	1.0298
Pf DG1	0.8665	0.8916
Pf DG2	0.9136	0.9008
P_curt_ DG1	19.18%	19.98%
P_curt_DG2	14.88%	4.34%

**Table 2:** Optimisation result with PSO and BSA for DGs with 2 MW generation of the 13-bus test system.

	PSO	BSA
Best fitness	0.0303	0.0298
Tap DG1	1.0052	0.9802
Tap DG2	1.0283	1.0298
Pf DG1	0.8678	0.9157
Pf DG2	0.9427	0.9254
P_curt_ DG1	15.37%	18.35%
P_curt_DG2	15.35%	8.430%

**Table 3:** Optimisation result with PSO and BSA for DGs with 3 MW generation of the 13-bus test system.

	PSO	BSA
Best fitness	0.0413	0.0407
Tap DG1	0.9912	0.9802
Tap DG2	1.0281	1.0298
Pf DG1	0.8721	0.8944
Pf DG2	0.9331	0.9136
P_curt_ DG1	15.85%	19.98%
P_curt_DG2	5.83%	0.94%

**Table 4:** DG overall impact on power loss, voltage deviation, and fitness for the three cases.

DG availability	Optimisation technique	Losses (MW)	Voltage deviation (%)	Fitness
No DG in the system		0.146	0.014	-
With 1 MW DG	PSO	0.0446	0.0017	0.0266
	BSA	0.0430	0.0016	0.0264
With 2 MW DG	PSO	0.0491	0.0015	0.0303
	BSA	0.0543	0.0009	0.0298
With 3 MW DG	PSO	0.0681	0.0018	0.0413
	BSA	0.0684	0.0006	0.0407

Tables 1 to 4 depict the results of case studies with DGs connected to the test system with different power generations. Results in Table 1 for the first case study show that the tap settings are approximately around 0.9935 and 1.0284 for the PSO technique, whereas tap settings are 0.9802 and 1.0298 for the BSA technique, On the other hand, the power factor settings for the BSA are approximately around 0.8916 and 0.9008 compared with 0.8665 and 0.9136 for the PSO. The total percentage of generation to be curtailed using PSO is approximately around 33%, whereas the percentage is around 24% using BSA. Power loss and voltage deviation are reduced by approximately 70% and greater than 87%, respectively for both techniques from the base case.

Results of the second case study are shown and summarised in Tables 2 and 4. The power factor settings using the BSA are around 0.9157 and 0.9254, while PSO recorded values of 0.8678 and 0.9427. Tap settings using the BSA technique are recorded as 0.9802 and 1.0298, while tap settings using PSO are approximately 1.0052 and 1.0283. The third controlled parameter which is percentage generation to be curtailed is 31% using PSO and 27% using BSA. This optimal combination successfully reduces the amount of losses to more than 60% for both techniques and reduces the voltage deviation to 89.28% and 93.57% using PSO and BSA, respectively.

The third case study also shows consistencies in the obtained results. The tap settings recorded using the BSA technique are 0.9802 and 1.0298 for the two DGs while the optimal tap setting values using PSO are recorded at 0.9912 and 1.0281. The power factors obtained for the DGs using BSA, which are 0.8944 and 0.9136, are still better compared with the PSO optimal settings of 0.8721 and 0.9331. For both optimisation techniques, the percentage of generation to be curtailed is approximately 20% and the amount of loss is reduced to more than 53%. The voltage deviation for this case study is reduced to 87.14% and 95.71% using PSO and BSA, respectively.

Table 4 shows that DGs installed with optimal parameter settings for the three coordinated voltage controls have significant influences on the reduction of total loss and voltage deviation in the distribution system. The optimal settings of the parameters using the BSA technique are more precise compared with the values obtained using the PSO technique. Results drawn from other works reviewed [26–27] show that the tap setting of approximately 1.02 p.u to 1.033 p.u can increase the amount of generation connected to the system and maintain the voltage within limits.

The optimal settings of power factor of the DGs recorded using BSA are nearly 0.90 for all the case studies. This finding is correlated with the settings of 0.90 as used by most distribution network operators in different countries, including Tenaga Nasional Berhad which is the public utility company that manages power generation and distribution in Malaysia. In the research conducted in [28], the maximum percentage of generation to be curtailed is ruled to be 40% of the percentage generation. Meanwhile, curtailing the generation by approximately 41% on a simple 7-bus test system can effectively mitigate voltage rise issue [26]. For all three case studies, the BSA technique recorded a lower amount of generation to be curtailed compared with that of the PSO.

**Fig 11:**
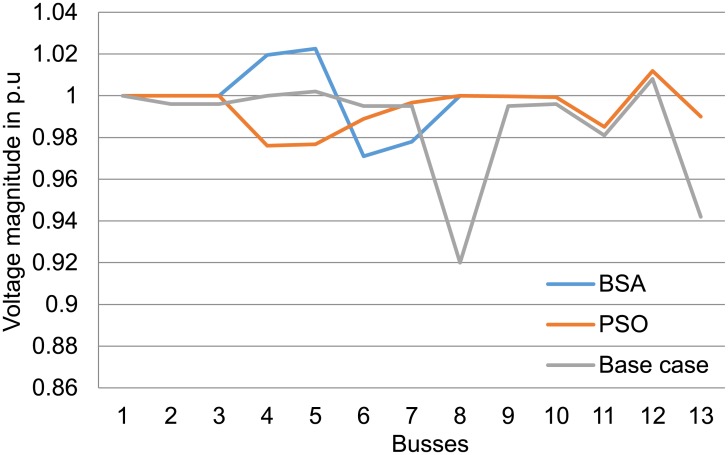
Voltage profile at the buses for the 13-bus system with 1 MW DGs using BSA and PSO.

**Fig 12:**
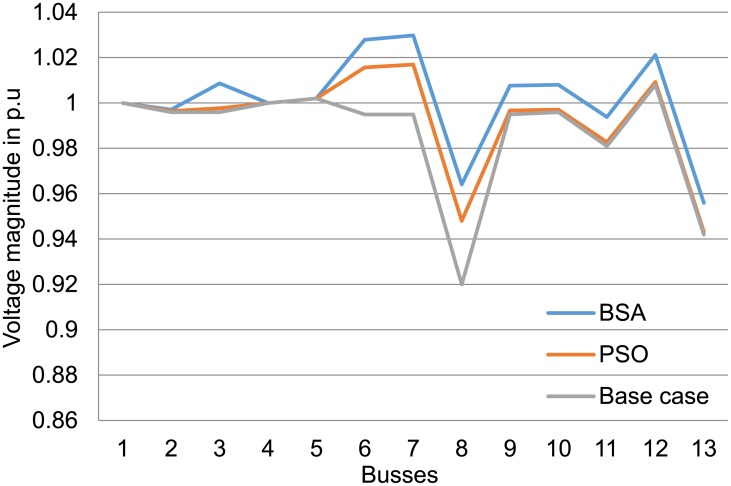
Voltage profile at the buses for the 13-bus system with 2 MW DGs using BSA and PSO.

**Fig 13:**
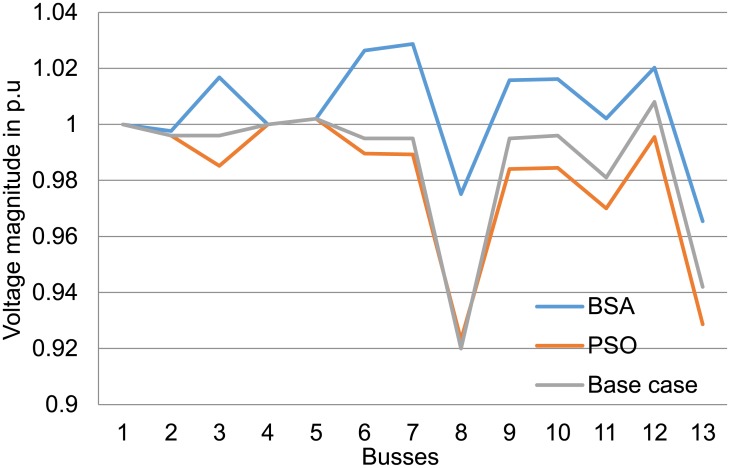
Voltage profile at the buses for the 13-bus system with 3 MW DGs using BSA and PSO.

Results in Table 4 also show that losses and voltage deviation are decreased dramatically when optimal settings of the voltage control are applied to the test system using both techniques. The BSA and PSO techniques have reduced voltage deviation at the load buses with BSA showing larger reduction compared with PSO in all the case studies. Both optimisation techniques have greatly reduced the percentage of losses to more than 53% for 3 MW DGs and more than 70% for the 1 MW case study. Figs 11 to 13 show the improvement in voltage profiles for the 13-bus test system before and after DG integration with optimal parameter settings. Findings from these figures illustrate that voltage profiles have improved using both PSO and BSA techniques, in which BSA optimisation controlled the voltage range between 0.98 and 1.02 p.u.

## Conclusion

This paper presents the application of the BSA which is a relatively new optimisation technique for determining the optimal settings of three different coordinated voltage control by means of PF, tap-changer and generation curtailment control. Results show that the BSA optimisation technique performs better in terms of the fitness function compared with the PSO technique. In terms of the optimal settings of the controlled parameters, the BSA has shown to obtain values that are more precise compared to that found in other works reviewed and guidelines used by other countries. The multi-objective function in terms of minimising losses and voltage deviation is also another important criterion that is successfully achieved. Tabulated results show that power losses are minimised in the range of 53% to 70% and that voltage deviation is minimised in the range of 87% to 95% for the three different case studies performed using both BSA and PSO techniques.
